# Characteristic of molecular subtypes in lung adenocarcinoma based on m6A RNA methylation modification and immune microenvironment

**DOI:** 10.1186/s12885-021-08655-1

**Published:** 2021-08-20

**Authors:** Hao Zhou, Miaosen Zheng, Muqi Shi, Jinjie Wang, Zhanghao Huang, Haijian Zhang, Youlang Zhou, Jiahai Shi

**Affiliations:** 1 Department of Thoracic Surgery, Affiliated Hospital of Nantong University and Medical School of Nantong University, Nantong, 226001 Jiangsu China; 2 Department of Pathology, Affiliated Hospital of Nantong University and Medical School of Nantong University, Nantong, 226001 Jiangsu China; 3 Medical School of Nantong University, Nantong, 226001 Jiangsu China; 4 Research Center of Clinical Medicine, Affiliated Hospital of Nantong University, Nantong, 226001 Jiangsu China

**Keywords:** Lung adenocarcinoma, m6A RNA methylation, Immune microenvironment, Prognostic model

## Abstract

**Background:**

Lung adenocarcinoma (LUAD) is a major subtype of lung cancer and closely associated with poor prognosis. N6-methyladenosine (m6A), one of the most predominant modifications in mRNAs, is found to participate in tumorigenesis. However, the potential function of m6A RNA methylation in the tumor immune microenvironment is still murky.

**Methods:**

The gene expression profile cohort and its corresponding clinical data of LUAD patients were downloaded from TCGA database and GEO database. Based on the expression of 21 m6A regulators, we identified two distinct subgroups by consensus clustering. The single-sample gene-set enrichment analysis (ssGSEA) algorithm was conducted to quantify the relative abundance of the fraction of 28 immune cell types. The prognostic model was constructed by Lasso Cox regression. Survival analysis and receiver operating characteristic (ROC) curves were used to evaluate the prognostic model.

**Result:**

Consensus classification separated the patients into two clusters (clusters 1 and 2). Those patients in cluster 1 showed a better prognosis and were related to higher immune scores and more immune cell infiltration. Subsequently, 457 differentially expressed genes (DEGs) between the two clusters were identified, and then a seven-gene prognostic model was constricted. The survival analysis showed poor prognosis in patients with high-risk score. The ROC curve confirmed the predictive accuracy of this prognostic risk signature. Besides, further analysis indicated that there were significant differences between the high-risk and low-risk groups in stages, status, clustering subtypes, and immunoscore. Low-risk group was related to higher immune score, more immune cell infiltration, and lower clinical stages. Moreover, multivariate analysis revealed that this prognostic model might be a powerful prognostic predictor for LUAD. Ultimately, the efficacy of this prognostic model was successfully validated in several external cohorts (GSE30219, GSE50081 and GSE72094).

**Conclusion:**

Our study provides a robust signature for predicting patients’ prognosis, which might be helpful for therapeutic strategies discovery of LUAD.

**Supplementary Information:**

The online version contains supplementary material available at 10.1186/s12885-021-08655-1.

## Introduction

Lung cancer is one of the common cancers worldwide, leading to high mortality every year [1]. Non-small cell lung cancer (NSCLC) accounts for 85% of cases, and lung adenocarcinoma (LUAD) is the most prevalent subtype [2, 3]. Despite great improvements in diagnostic and therapeutic techniques, the prognosis for LUAD patients is still poor [4]. Therefore, identifying treatment targets and effective predictors to improve the prognosis of LUAD patients is critical.

N6-methyladenosine (m6A) is one of the most common mRNA internal modifications in eukaryotic organisms and methylation modificatied at the N6 position of adenosine residues in RNA [5, 6]. The modification of RNA m6A is involved in many essential biological processes including gene expression, immunomodulation and cancers [7]. In addition, RNA modifications, especially m6A modification, has been proved to be necessary for tumor development [8]. The tumor microenvironment (TME) is important in the formation, development, and treatment of tumors which contains tumor cells, immune cells and stromal cells [9]. Tumor infiltrating immune cells are an important component of the complex microenvironment. Currently, TME has been the hot pot of the tumor field because research suggested that TME immune cells are closely related to the prognosis and malignancy of tumors [10]. Hence, exploring the relationship between m6A RNA methylation and immune microenvironment is beneficial to improve the prognosis accuracy of patients with LUAD.

In this study, we assessed the expression of 21 m6A regulatory factors from TCGA database, and the TCGA patients were divided into two clusters according to the expression of these genes. Then, a risk prognostic signature was established on the base of the differentially expressed genes (DEGs) between the two clusters. We analyzed and compared the different immune cell infiltration and clinical outcomes between the high-risk group and low-risk group. Furthermore, this prognostic model showed pretty good predictive accuracy compared with other clinical factors. Importantly, we validated the prognostic model in three independent external cohorts (GSE30219, GSE50081 and GSE72094). These results discovered novel insight for the diagnosis and treatment of LUAD by using bioinformatics tools.

## Materials and methods

### Data source

The TCGA database was used to obtaine the gene expression profile cohort and its corresponding clinical data of LUAD patients, including the FPKM value of gene expression of 535 LUAD samples and 59 normal samples. Four hundred ninety-four samples with complete survival information were used for subsequent analysis. Because a considerable number of patients lack the clinical information of M classification, in order to ensure the number of samples, we did not include M classification in the clinical correlation analysis. The normalized matrix files of the three cohorts (GSE30219, GSE50081 and GSE72094) from the GEO database were downloaded for the validation data sets.

### Consensus cluster analysis for 21 m6A regulators

In this study, we included 21 m6A regulators including 8 writers (METTL3, METTL14, RBM15, RBM15B, WTAP, VIRMA, CBLL1, ZC3H13), 2 erasers (ALKBH5, FTO) and 11 readers (YTHDC1, YTHDC2, YTHDF1, YTHDF2, YTHDF3, IGF2BP1, HNRNPA2B1, HNRNPC, FMR1, LRPPRC, ELAVL1) [11]. Based on the expression of 21 m6A regulators, we performed consensus classification to identify different m6A modification patterns. The patients were divided into different subtypes using the R package “ConsensusClusterPlus” for further analysis. To ensure the stability of classification, 1000 iterations, and a resample rate of 80% were conducted. The cumulative distribution function (CDF) curve was used to determine the clustering number [12].

### Inference of tumor microenvironment and immune cells

The immune score was calculated by applying the ESTIMATE algorithm to each patient via the “estimate” R package [13]. The single-sample gene-set enrichment analysis (ssGSEA) algorithm was conducted to quantify the relative abundance of the fraction of 28 immune cell types [14].

### Differential gene expression and functional analyses

Wilcoxon test conducted by R software was performed to identify the differentially expressed genes between cluster 1 and cluster 2. The cut-off criteria was | logFC | > 1 and FDR < 0.05. Gene ontology (GO) enrichment analysis by clusterProfiler R package was performed to know the potential biological processes associated with the DEGs [15]. The *P*-value adjusted < 0.05 was regarded significant.

### Risk assessment model construction

Firstly, 457 DEGs were identified between two clusters for our data screening. The DEGs associated with overall survival (OS) were screened via univariate Cox regression analysis. Then, Lasso Cox regression was used to selected the most striking markers to establish the prognostic model. The optimal value of the penalty parameter λ was defined by 10-fold cross validation [16]. The risk score was obtained after multiplying the expression level of each gene by its coefficient obtained from the LASSO Cox regression. Then, the patients were divided into high-risk and low-risk groups according to the optimal cut-off value.

### Gene set enrichment analysis

GSEA was used to investigate potential biological functions between different risk groups of LUAD patients [17]. The number of random sample permutations was performed 1000 times. The significance was based on the false discovery rate (FDR) < 0.05 and *P*-value < 0.05.

### Statistical analysis

All data analyses were conducted using R language (version 3.6.3). Kaplan-Meier method was used to draw survival curves. The receiver operating characteristic (ROC) curve was plotted with the SurvivalROC R package. The Wilcoxon test was used to compare the two groups’ differences. The correlation tests were conducted by Pearson correlation analysis. Univariate and multivariate analyses were conducted to determine whether the prognostic model was independently variable when integrated with other clinical factors. Statistical *P* < 0.05 was considered significantly.

## Results

### The different expression of m6A RNA methylation regulatory molecules and consensus clustering analysis

**Fig. 1:**
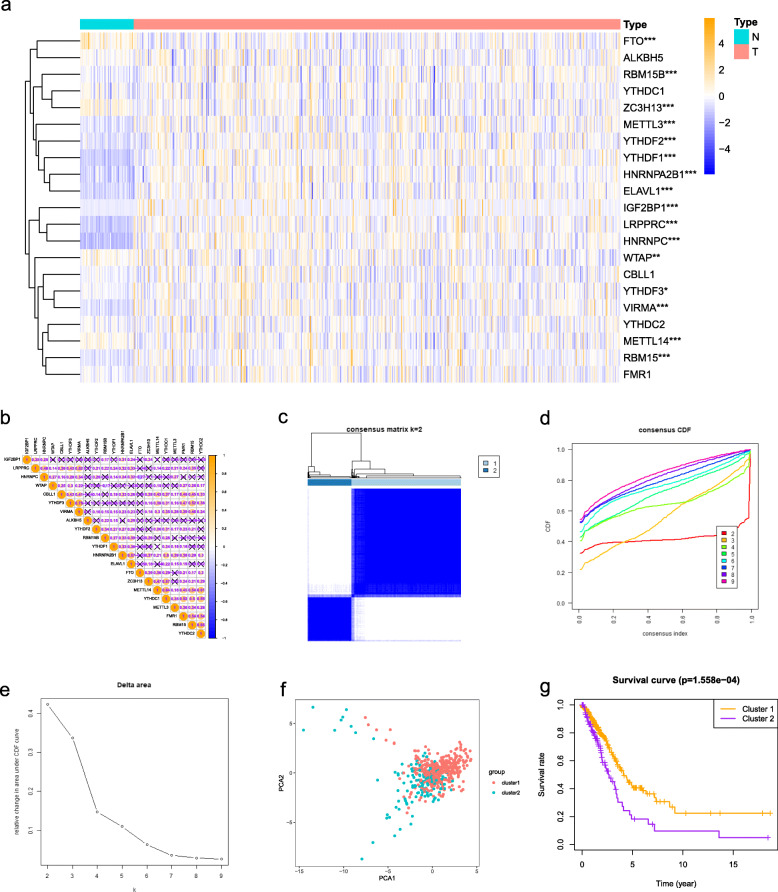
Expression of m6A RNA methylation regulators and consensus clustering analysis. **a** The heat map shows the expression of 21 m6A regulatory factors between LUAD and adjacent normal tissues. **b** The co-expression patterns between 21 m6A regulatory factors. **c** Consensus clustering of LUAD patients for k = 2. **d** Consensus clustering CDF for k = 2–9. **e** The CDF curve of consensus clustering. **f** Principal component analysis of the mRNA expression profile in LUAD patients. **g** The prognostic analysis between cluster 1 and cluster 2. *p < 0.05, **p < 0.01, and ***p < 0.001

In order to explore the potential role of m6A regulatory factors in the occurrence and development of LUAD, we systematically analyzed the expression of 21 m6A regulatory factors between LUAD and adjacent normal tissues. The different expression values of these genes between LUAD and normal tissues were observed in Fig. 1a. Figure 1b showed the important co-expression patterns between all the 21 m6A regulatory factors. The results indicated that YTHDF3 was strongest positively correlated with VIRMA (*r* = 0.75). RBM15 was positively associated with YTHDC2 (*r* = 0.65). METTL14 was positively associated with YTHDC1 (*r* = 0.64). Instead, FTO was negatively associated with HNRNPC (*r* = − 0.27). Then, we performed a consensus classification to determine the optimal number of clusters by looking for a suitable k value. The k = 2 was demonstrated to have the best clustering stability (Fig. 1c-e). The LUAD patient cohort was separated into two clusters, namely cluster 1 and cluster 2. In addition, PCA shows two distinct distribution patterns, which shows that the classification generated by consensus clustering analysis is effective (Fig. 1f). The prognostic analysis showed that the OS of patients with LUAD in cluster 2 was obviously shorter than that in cluster 1 (Fig. 1g).

### Tumor immune microenvironment of the two clusters of LUAD patients

**Fig. 2:**
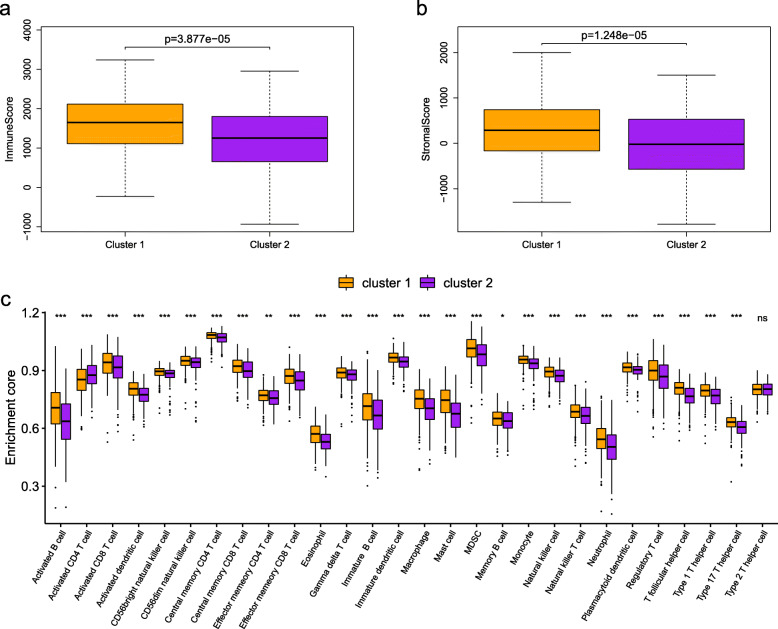
Tumor immune microenvironment between cluster 1 and cluster 2 patients with LUAD. The difference of overall **a** Immune scores and **b** stromal scores between cluster 1 and cluster 2 patients. **c** Differences in 28 immune infiltration cells between cluster 1 and cluster 2

A piece of increasing evidence indicated the close relationship between the malignant degree of cancers and the tumor immune microenvironment. Considering the obvious differences of the OS in two clusters of LUAD patients, we speculated that tumor immune microenvironment might be an important contributor to the LUAD progress. Then we explored the differences of immune infiltration to distinguish the patients in two clusters. Utilizing the ESTIMATE algorithm, we found that the immune score of cluster 1 was much higher than that of cluster 2 (Fig. 2a). And the stromal score presented the same tendency (Fig. 2b). Based on the ssGSEA, we evaluated the proportion difference of 28 immune cell types in the two clusters (Table S1). As shown in Fig. 2c, the two clusters had significantly different characteristics of immune cell infiltration. In addition, the degree of immune cell infiltration in cluster 1 was significantly higher than that in cluster 2. Overall, these findings demonstrated that the presence of different immune cell populations may influence the OS of the patients with LUAD.

### Identification of differentially expressed genes and functional analyses

**Fig. 3:**
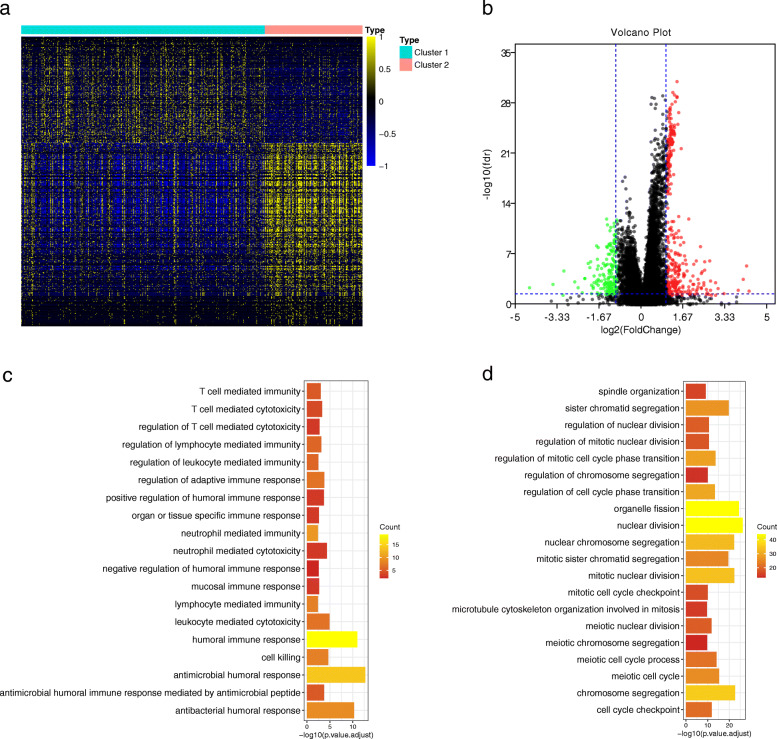
Identification of differentially expressed genes between two clusters. **a** The heat map and **b** volcano plot of differentially expressed genes between cluster 1 and cluster 2. **c** GO enrichment analyses of the genes up-expressed in cluster 1. **d** GO enrichment analyses of the genes up-expressed in cluster 2

Four hundred fifty-seven DEGs were identified to further explore the differences between the two m6A modification patterns (Table S2). Among them, 168 DEGs were up-regulated in cluster 1 and 289 DEGs were up-regulated in cluster 2. Figure 3a-b showed the heat map and volcano plot. For better understanding the biological functions of the two clusters based on DEGs, GO enrichment analysis was performed by clusterProfiler R package. And it was found that overexpression of genes in cluster 1 mainly enriched in immune-related biological processes, such as humoral immune response and regulation of adaptive immune response (Fig. 3c and Table S3). The genes overexpressed in cluster 2 were found to be mainly enriched in many biological processes related to the cell cycle (Fig. 3d and Table S4).

### Construction of a seven-gene prognostic model

**Fig. 4:**
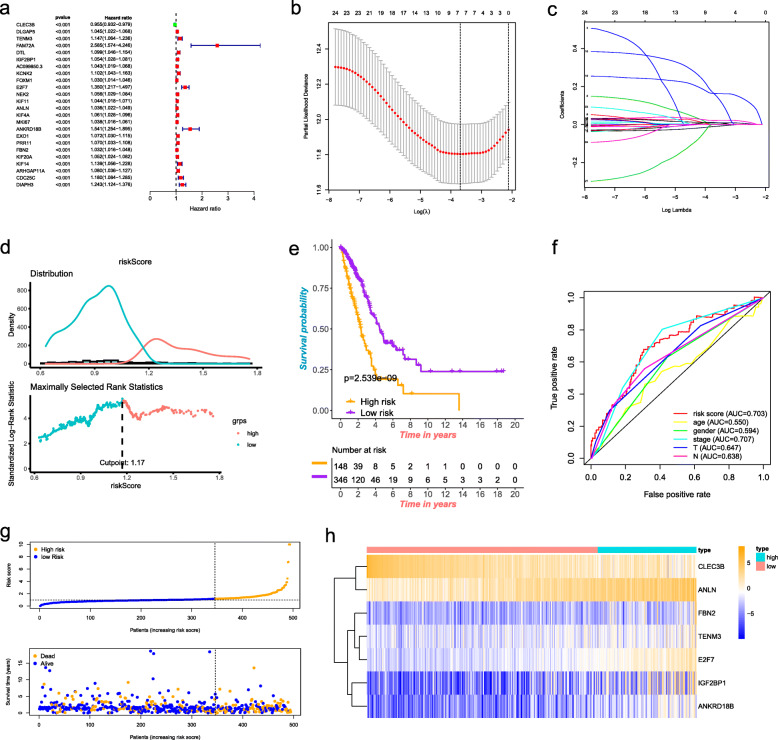
Construction of a senen-gene prognostic model. **a** Survival-associated genes and forest plot. **b** Tenfold cross-validation for tuning parameter selection in the Lasso model. **c** Lasso coefficient profiles of the 24 survival-associated genes. **d** The optimal cut-off value to separate patients into high- and low-risk groups. **e** Survival analyses for patients in high- and low-risk groups. **f** ROC curve analysis based on TCGA cohort. **g** The risk score distribution and the survival status. **h** Expression heat map of the seven key genes

With univariate Cox regression analysis, 24 prognostic-related genes based on the DEGs were identified (Fig. 4a). Then, Lasso Cox regression was conducted to determine the key genes with the best prognostic value by reducing the dimensionality and calculate the relative coefficients of the genes (Fig. 4b-c). Ultimately, seven optimal genes including CLEC3B, TENM3, IGF2BP1, E2F7, ANLN, ANKRD18B and FBN2 were chosen to construct the prognostic model for LUAD. The coefficients for each gene were listed in Table S5. The risk score for each patient was acquired by multiplying the each gene’s expression level by its coefficient. Then, according to the optimal cut-off value, the patients were divided into high- and low-risk groups (Fig. 4d). One hundred forty-eight patients were in the high-risk group and 346 patients were in the low-risk group. Besides, the survival analysis in Fig. 4e revealed the worse prognosis in patients with a high-risk score. The sensitivity of the prognostic model was assessed by ROC curve (Fig. 4f). And the AUC result of this risk score model was 0.703. Compared with other clinical parameters, our prognostic risk signature had pretty good predictive accuracy. The risk score distribution and each patient’s survival status were shown in Fig. 4g. The tendency of seven hub genes’ expression in high-risk and low-risk groups was shown in the heat map of Fig. 4h.

### Correlation analysis was performed between the risk model and immune cell infiltration

**Fig. 5:**
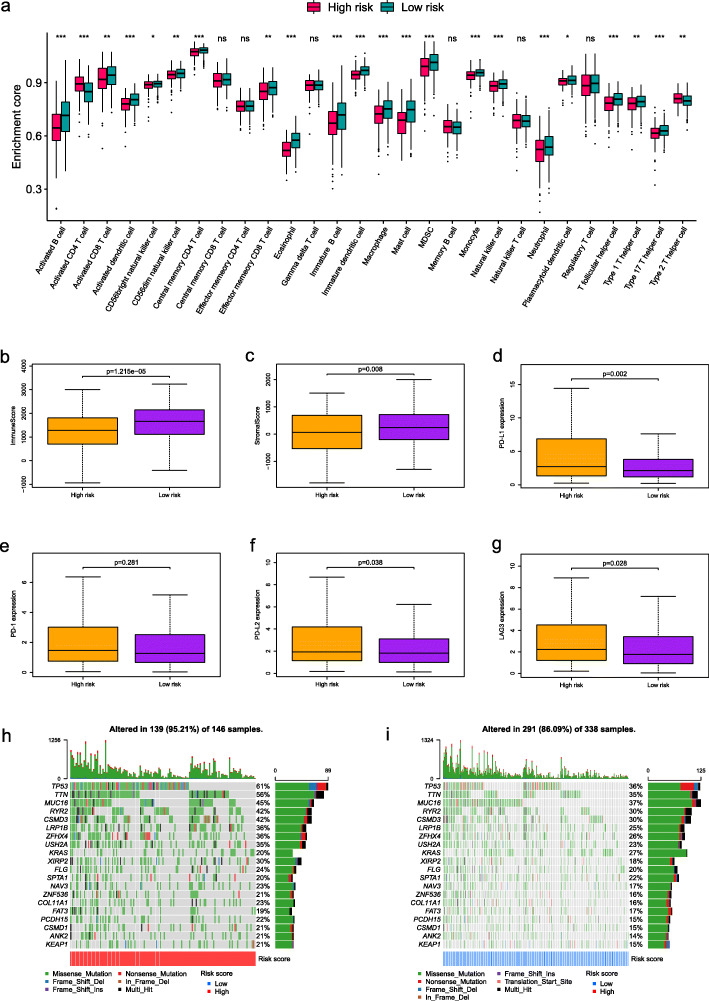
Correlation analysis of the prognostic model and immune cell infiltration. **a** Differences in 28 immune infiltration cells between high- and low-risk groups. **b** Immune scores and **c** stromal scores between high- and low-risk patients. The expression level of immune checkpoint molecules including **d** PD-L1, **e** PD-1, **f** PD-L2, and **g** LAG3 between high- and low-risk patients. The waterfall plot showing the differences of tumor somatic genomic mutation between **h** high-risk group and **i** low-risk group

To explore the difference of tumor immune microenvironment between the two groups with different degrees of risk, the infiltration levels of 28 immune cell types were evaluated based on ssGSEA (Fig. 5a). The result suggested that high immune cell infiltration in a significant number of low-risk samples. Then, we examined the distribution of stromal and immune scores between the two risk groups of patients using the ESTIMATE algorithm. Comparing with the high-risk group, the low-risk group presented higher immune and stromal scores (Fig. 5b-c). In addition, PD-L1, PD-1, PD-L2, and LAG3 were assessed to know these different expression of immune checkpoint molecules in different groups (Fig. 5d-g). When compared to patients in low-risk group, high-risk group patients had higher expression of immune checkpoint molecules. This indicated that the prognosis model may have a potential role in predicting patients’ response to anti-checkpoint immunotherapy. Based on the TCGA cohort, we further analyzed the distribution differences of somatic genomic mutation between high-risk group and low-risk group. We found that high-risk group presented more extensive tumor mutation burden than low-risk group (Fig. 5h-i). In addition, we analyzed the difference in miRNA of the two groups based on the risk score. A total of 53 differentially expressed miRNAs were identified with the cut-off criteria | logFC | > 1 and FDR < 0.05 based on the TCGA cohort. The results were shown in Table S6 and visualized in the form of volcano plot in Fig. S1.

### Correlation assessment between risk signature and clinical features

**Fig. 6:**
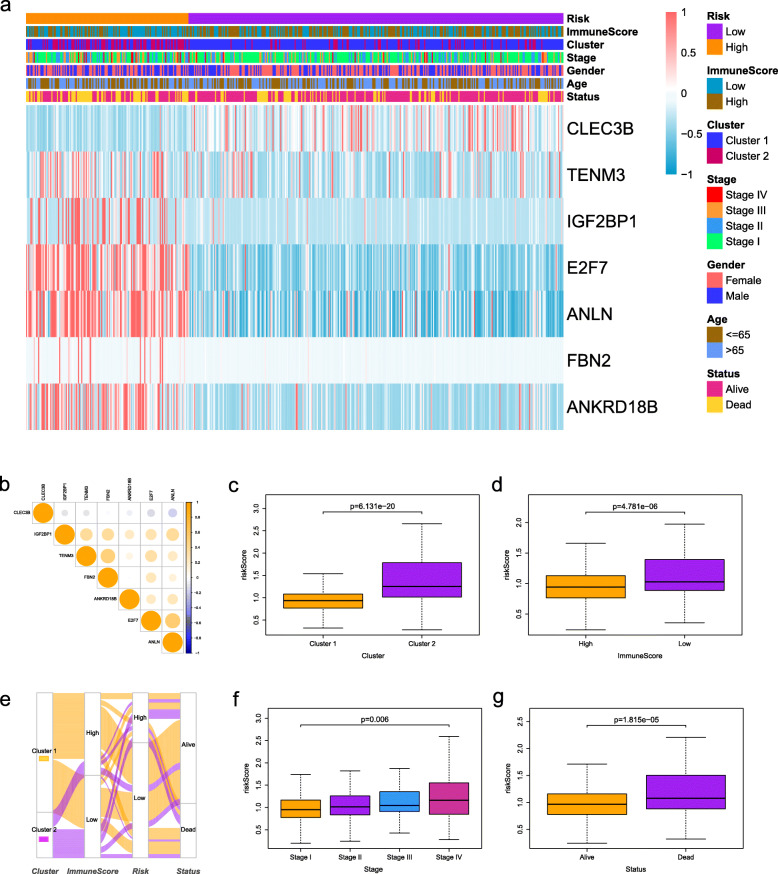
Correlations between the risk signature and clinical features. **a** The heat map and clinicopathologic features of the two clusters. **b** The relationship between the seven key genes. Distribution of risk scores stratified by **c** cluster, **d** immunoscore, **f** stage, and **g** status. **e** The alluvial diagram shows the attribute changes of individual patients

We further analyzed the relationship between the risk score model and clinical features. Figure 6a summarized the expression levels of the seven key genes as a heat map. Almost all these genes expressed highly in the high-risk group while CLEC3B highly expressed in the low-risk group. In addition, CLEC3B was also highly expressed in normal samples and related to better OS while the rest six genes were highly expressed in tumor samples and associated with worse OS (Fig. S2-S3). The correlation between the seven key genes was showed in Fig. 6b. The high-risk and low-risk groups have a significant difference in terms of stages, status, clustering subtypes, and immunoscore (Fig. 6a). Compared to the cluster 2, cluster 1 had a lower risk score (Fig. 6c). This was also in line with our previous result that patients in cluster 1 had a better OS. Moreover, compared to the low immunescore group, a lower risk sore in the high immunescore group was observed in Fig. 6d. With the clinical-stage increased, the risk score also increased (Fig. 6f). Figure 6g revealed a slightly better status in the low-risk group patients. These findings demonstrated that the prognostic risk signature was closely related to the malignant degree of tumor. And the attribute changes of individual patients can be clearly presented by an alluvial diagram (Fig. 6e).

**Fig. 7:**
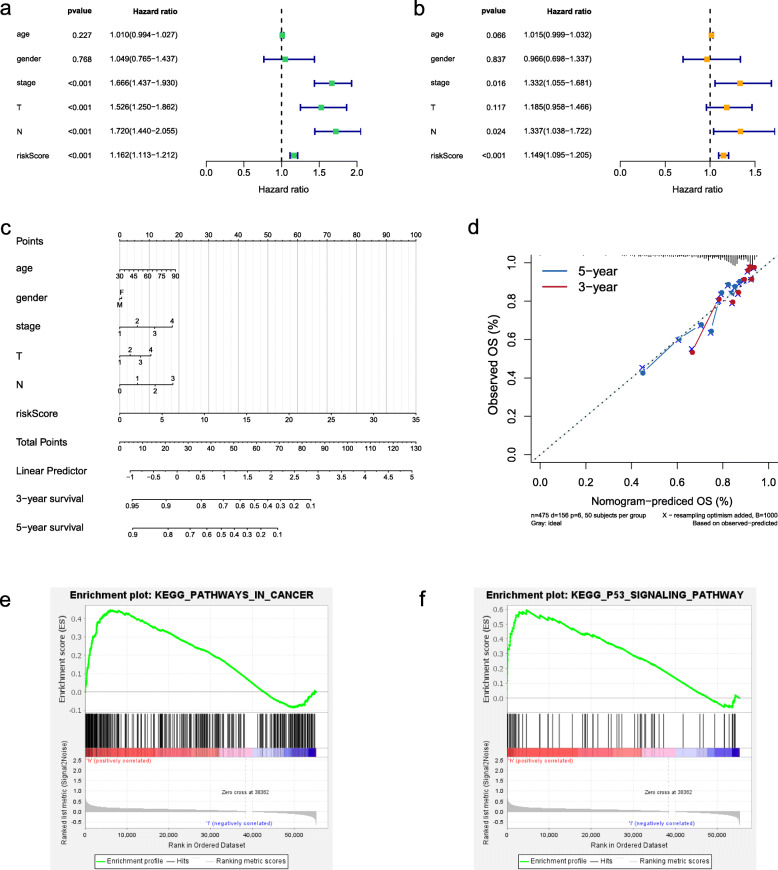
Prognostic value of the senen-gene prognostic model. **a** Univariate and **b** multivariate cox regression analysis of clinical characteristics and the prognostic model. **c** A nomogram was constructed by combining clinicopathologic characteristics and risk score. **d** Calibration plots of the nomograms for predicting 3-year, and 5-year outcomes. **e-f** Two significant pathways activated in high-risk groups were identified using GSEA enrichment analysis

Considering other clinical features in TCGA, whether the risk score for OS is an independent indicator needs further exploration. As the univariate analysis shown in Fig. 7a, the clinical stage, T stage, N stage, and risk score were related to poorer survival. The multivariate analysis indicated that the risk score could be considered as an independent prognostic factor for patients with LUAD (Fig. 7b). To facilitate the utilization of risk score, a nomogram was plotted considering risk score and other clinical factors (Fig. 7c). Calibration plots for 3-year and 5-year OS were used to visualize the performances of the nomograms (Fig. 7d). To further explore the biological pathways associated with the risk signature, we performed GSEA enrichment analysis and found that pathways in cancer and p53 signaling pathway were activated in high-risk groups (Fig. 7e-f).

### Validation of the prognostic signature by the GEO database

**Fig. 8:**
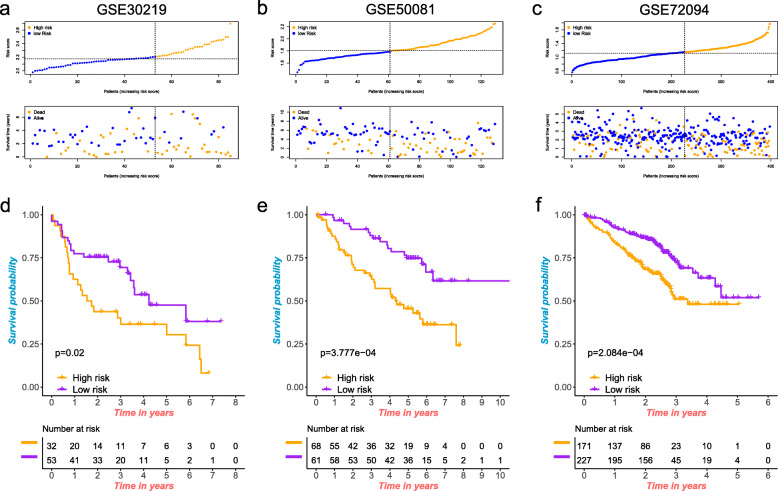
Validation of the prognostic model utilizing external cohorts. **a-c** The risk score distribution and survival status of the patients in the three cohorts (GSE30219, GSE50081 and GSE72094). **d-f** Survival curves for the high- and low-risk groups in the three cohorts (GSE30219, GSE50081 and GSE72094)

To determine the prognostic potential of the seven-gene signature in other datasets, Three GEO data sets (GSE30219, GSE50081 and GSE72094) were adapted as independent external validation. The same formula was used to calculate the risk score for each patient in the GEO cohorts. Then, according to the optimal cut-off value, we divided LUAD patients into high-risk group and low-risk group. The patients’ risk score distribution and survival status were shown in Fig. 8a-c. The survival curves revealed that patients in the high-risk group have a shorter survival time than patients in the low-risk group (Fig. 8d-f). Therefore, our risk signature can well distinguish patients according to the risk score. It is helpful for prognosis prediction and treatment of LUAD patients.

## Discussion

Lung cancer is a global public health challenge with its high mortality [18]. LUAD, a fatal malignancy associated with poor prognosis and high mortality rates, accounts for more than 40% of the total incidence of lung cancer [19, 20]. Although the diagnosis and treatment of LUAD have made great progress, the effective diagnosis and prognosis prediction of LUAD patients is still a major clinical challenge [21]. Therefore, identifying key molecules and constructing a prediction model with high stability and effectiveness are conducive to the implementation of precise treatment and improve the prognosis of patients.

N6-methyladenosine (m6A) is the most abundant internal modification of eukaryotic mRNA. Almost every stage of mRNA metabolism is affected by m6A mRNA methylation [22]. In addition, new evidence suggests that m6A RNA methylation plays a vital role in tumorigenesis and development [23]. FTO and METTL3 have been reported as potential targets for the diagnosis and treatment of LUAD patients. It is reported that FTO facilitates LUAD cell progression by activating cell migration through m6A demethylation [24]. High expression of METTL3 in LUAD is believed to promote the growth and invasion of cancer cells [25]. However, there are few studies on the relationship between m6A related genes and LUAD. In this study, the expression of 21 m6A regulatory factors from LUAD and adjacent normal tissues were systematically analyzed. We observed the different expression levels of these genes between LUAD and normal tissues. Based on the expression of 21 m6A regulatory factors, TCGA patients were divided into two clusters utilizing consensus classification. The prognosis of patients in cluster 1 was better than that of patients in cluster 2. Given that the patients’ prognosis was probably related to tumor immune microenvironment, the differences of immune infiltration between two clusters were analyzed. We found that patients in cluster 1 had a higher immune score and immune cell infiltration. Previous studies indicated that the immune-inflamed phenotype shows the infiltration of massive amounts of immune cells in the tumor microenvironment, and correlated with better prognosis [11, 26]. To further explore the difference between the two clusters, we identified 457 DEGs and analyzed their biological functions by GO enrichment analyses. Interestingly, the immune-related biological processes were mainly enriched for the up-expressed genes in cluster 1. Then, after selecting seven survival-associated DEGs by Lasso Cox regression, a reliable prognostic model was successfully established. The survival analysis demonstrated patients with a high-risk score have a worse prognosis. The ROC curve confirmed the predictive accuracy of this prognostic risk signature. In addition, higher immune scores and immune cell infiltration were founded in the low-risk group. In recent years, immune checkpoint inhibitors have attracted much attention due to their promising application in the immunotherapy of cancer [27]. Therefore, expressions of immune checkpoint molecules like PD-L1, PD-1, PD-L2, and LAG3 in different groups were examined. And it was found that there existed a positive correlation between risk score and the expressions of immune checkpoint molecules. Therefore, these results demonstrated that the prognosis model might have a potential role in predicting the clinical response of immunotherapy.

Among the seven key molecules (CLEC3B, TENM3, IGF2BP1, E2F7, ANLN, ANKRD18B, and FBN2), only CLEC3B highly expressed in the low-risk group while other genes highly expressed in the high-risk group. CLEC3B, C-type lectin domain family 3 member B, is a member of the C-type lectin superfamily [28]. It encodes tetranectin, a plasminogen kringle-4-binding protein in cells [29]. It is reported that CLEC3B was down-regulated in several tumors and considered as a tumor suppressor in oral squamous cell carcinoma [30]. A previous study demonstrated that the expression of CLEC3B is correlated with the level of immune infiltration in lung cancer, and it is promising to be the important marker for the early diagnosis of lung cancer [31]. The protein encoded by TENM3 gene belongs to the teneurin family and is involved in tumorigenesis and drug resistance [32]. It is reported that TENM3 was up-regulated in tumor tissues, and it may function as an oncogenic gene in esophageal cancer [33]. However, the role of TENM3 in lung cancer is not yet clear. IGF2BP1 is an RNA-binding protein that participates in tumor progression, tumor cell proliferation and growth [34, 35]. The let-7 family exerts its role in suppressing the migration and growth of tumor cells by inhibiting the expression of IGF2BP1 [36]. Previous studies showed the up-regulation of IGF2BP1 in LUAD, which affects the progression of the disease [37, 38]. Furthermore, high expression of IGF2BP1 was associated with poor OS in LUAD [37]. E2F7 is a member of the E2F transcription factors family. It is reported that the mammalian E2F transcription factors play vital roles in the cell cycle, so they are closely related to cancer [39]. In addition, E2F7 has been found up-regulated in various malignant tumors, such as acute myeloid leukaemia and cutaneous squamous cell carcinomas [40, 41]. Knockdown of E2F7 can repress cell growth in endometrial carcinoma [42]. However, how E2F7 participates in LUAD is still unknown. The ANLN gene encodes an actin-binding protein that contributes to cell growth and migration [43]. The expression of ANLN is up-regulated in a variety of types of tumors, including lung cancer, and the development of cancers is related to the expression level of ANLN [44]. Previous studies indicated the vital role of ANLN in cell proliferation, and lacking ANLN reduced cell migration and invasion [45, 46]. ANLN has been reported to participate in the metastasis of LUAD by promoting the EMT of tumor cells [47]. ANKRD18B is a member of ANKRD family that functions in the occurrence of cancer, evidence that over-expression of ANKRD18B suppressed the growth of lung cancer cells has been reported [48]. FBN2 encoded the protein which belongs to the connective tissue microfibrils and participates in elastic fiber assembly. Nevertheless, there is no doubt to determine the impact of FBN2 in LUAD in the future.

Nevertheless, our study had a few limitations to be considered. Firstly, further experiments are necessary to verify our results because our study was only based on public databases. Secondly, larger sample size is necessary to confirm the predictive ability of our prognostic models. Thirdly, the biological roles of the seven key genes in LUAD require further experimental validation.

## Conclusions

In summary, based on the differentially expressed genes between the two clusters, a reliable prognostic model was established and identified as an independent prognostic predictor for LUAD. Furthermore, this risk signature could also be considered as a predictor of increased immune cell infiltration, proving its potential role in the tumor immune microenvironment. Importantly, the prognostic value of this risk signature was successfully validated in independent external cohorts (GSE30219, GSE50081 and GSE72094). Our current study provides a robust prognostic model to predict the prognosis of LUAD patients, which may provide significant guidance for the diagnosis and treatment of LUAD.

## Supplementary Information

**Additional file 1: Table S1.** Estimating relative abundance of 28 different immune cell types infiltration by the Single-Sample Gene-Set Enrichment Analysis (ssGSEA). **Table S2.** The differentially expressed genes between cluster 1 and cluster 2. **Table S3.** Gene Ontology (GO) enrichment analyses for differentially expressed genes up-regulated in cluster 1. **Table S4.** Gene Ontology (GO) enrichment analyses for differentially expressed genes up-regulated in cluster 2. **Table S5.** Regression coefficients of the genes in the prognostic model. **Table S6.** The differentially expressed miRNAs between high risk group and low risk group.

**Additional file 2: Figure S1.** The volcano plot of differentially expressed miRNAs between high risk group and low risk group.

**Additional file 3: Figure S2.** Survival analyses for the seven key genes between LUAD and adjacent normal samples. (a) CLEC3B, (b) TENM3, (c) IGF2BP1, (d) E2F7, (e) ANLN, (f) ANKRD18B, (g) FBN2.

**Additional file 4: Figure S3.** Paired sample expression analyses of the seven key genes. (a) CLEC3B, (b) TENM3, (c) IGF2BP1, (d) E2F7, (e) ANLN, (f) ANKRD18B, (g) FBN2.

## Data Availability

The datasets downloaded for supporting the results of this article are publicly available at the TCGA (https://portal.gdc.cancer.gov/) and GEO (https://www.ncbi.nlm.nih.gov/geo/).

## Abbreviations

LUAD: Lung adenocarcinoma
m6A: N6-methyladenosine
DEG: Differentially expressed gene
NSCLC: Non-small cell lung cancer
TME: Tumor microenvironment
CDF: Cumulative distribution function
ssGSEA: single-sample gene-set enrichment analysis
GO: Gene ontology
OS: Overall survival
FDR: False discovery rate
ROC: Receiver operating characteristic
GEO: Gene Expression Omnibus
LASSO: Least absolute shrinkage and selection operator
TCGA: The Cancer Genome Atlas
