# Supplementary figures and images for: Characteristic of molecular subtypes in lung adenocarcinoma based on m6A RNA methylation modification and immune microenvironment
Source: BMC Cancer. 2021 Aug 20;21:938. doi: 10.1186/s12885-021-08655-1 (PMC8379743; doi:10.1186/s12885-021-08655-1)

Volcano Plot

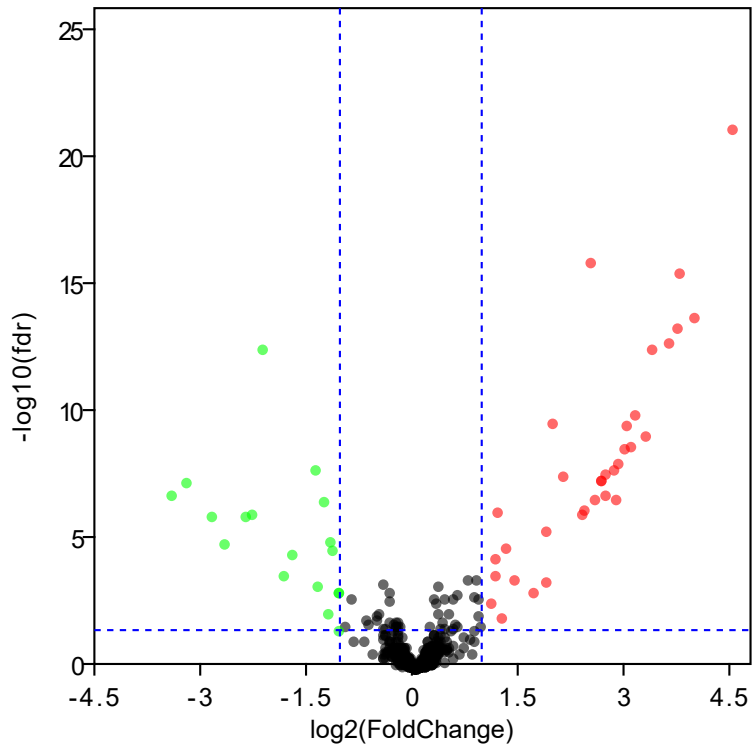

Supplement: Supplementary file 2 — Additional file 2: Figure S1. The volcano plot of differentially expressed miRNAs between high risk group and low risk group. [file 12885_2021_8655_MOESM2_ESM.pdf]

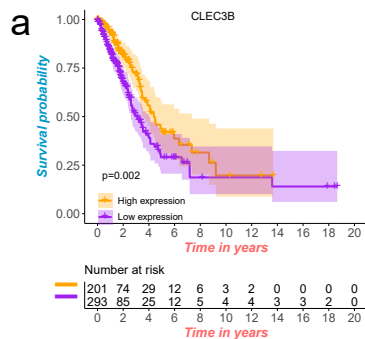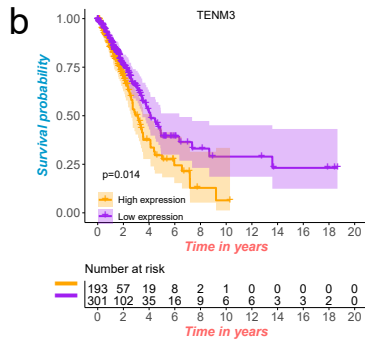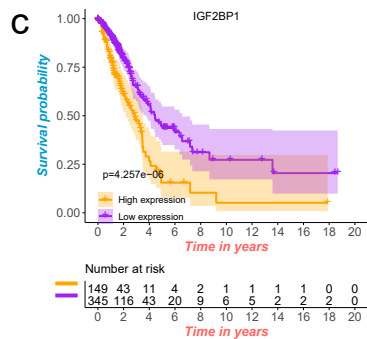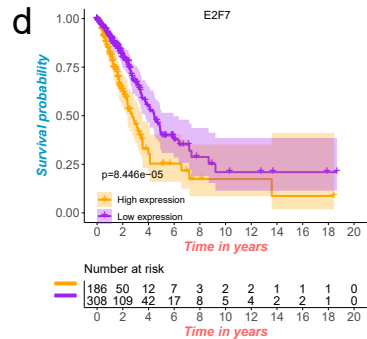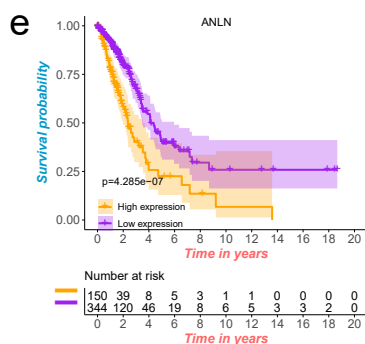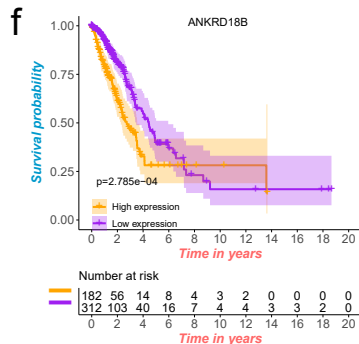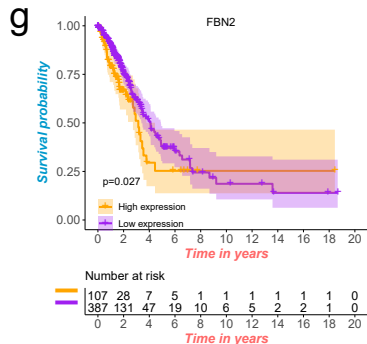

Supplement: Supplementary file 3 — Additional file 3: Figure S2. Survival analyses for the seven key genes between LUAD and adjacent normal samples. (a) CLEC3B, (b) TENM3, (c) IGF2BP1, (d) E2F7, (e) ANLN, (f) ANKRD18B, (g) FBN2. [file 12885_2021_8655_MOESM3_ESM.pdf]

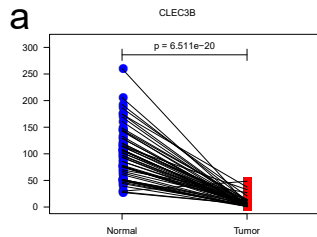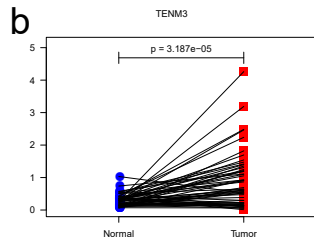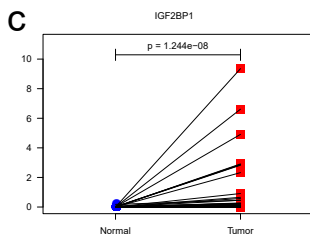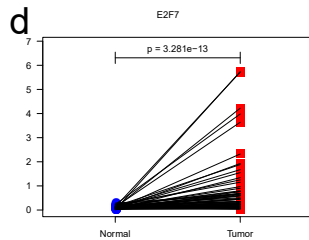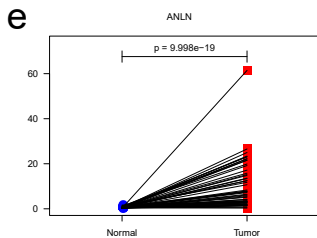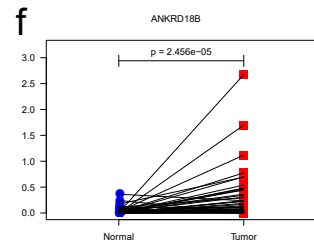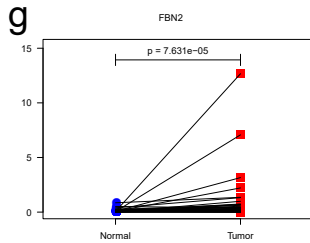

Supplement: Supplementary file 4 — Additional file 4: Figure S3. Paired sample expression analyses of the seven key genes. (a) CLEC3B, (b) TENM3, (c) IGF2BP1, (d) E2F7, (e) ANLN, (f) ANKRD18B, (g) FBN2. [file 12885_2021_8655_MOESM4_ESM.pdf]
